# Time, valence, and imagination: a comparative study of thoughts in restricted and unrestricted mind wandering

**DOI:** 10.1007/s00426-024-01969-2

**Published:** 2024-05-20

**Authors:** Halleyson Li, Thomas Hills

**Affiliations:** Department of Psychology, University of Warwick, Coventry, UK

## Abstract

William James’ “stream of thought” is a key component of human cognition. Such thoughts arise in both restricted and unrestricted contexts, either with or without the presence of a secondary task. This study examines the similarities and differences in thoughts produced in these two contexts, which we call restricted and unrestricted mind wandering. Participants performed a mindfulness task representing restricted mind wandering and an unrestricted thought task where they spontaneously explored thoughts, reporting them as they arose. Participants then self-rated their thoughts based on valence, temporal orientation (past/present/future), and reality orientation (imaginary vs. real). Participants’ emotional states were also evaluated using the Emotion Recall Task (ERT) and the PANAS questionnaire. Unrestricted mind wandering generated more thoughts, which were more positive and future-oriented than those in restricted mind wandering. Additionally, participants’ thought valence correlated with their PANAS and ERT scores. Approximately 1 out of 4 thoughts in both restricted and unrestricted mind wandering were imaginary, with increased future orientation linked to more imaginative thought. Despite the statistical differences separating restricted and unrestricted thought, effect sizes were predominantly small, indicating that the thoughts arise during these two types of mind wandering are largely of the same kind.

## Introduction

In the realm of conscious awareness, we navigate through diverse thought landscapes, continuously moulded by our interactions with our internal and external worlds (Mildner & Tamir, 2019; Smallwood, 2013). This phenomenon, what William James called our “stream of thought” occupie^2018^s the majority of our waking life and is considered a key component of the brain’s default process (Buckner et al., 2008; James, 2007; Killingsworth & Gilbert, 2010; Smith et al., 2018 ). Recent decades have seen a burgeoning interest in understanding the nature of this ‘stream’, its contents, and implications (i.e., Belardi et al., 2022; Callard et al., 2013; Krans et al., 2015; Sekiguchi, 2023; Seli, Risko, Smilek, & Schacter, 2016a; Smallwood & Schooler, 2006; Smith et al., 2018). This self-generated mental activity also goes by a variety of names and encompasses a variety of phenomena, including mind wandering, daydreaming, task-unrelated thought, spontaneous thought, stimulus-independent thought, fantasy, and imagination (Callard et al., 2013; Mildner & Tamir, 2019; Seli et al., 2018). A critical distinction among these phenomena is whether the thought occurs in a restricted or unrestricted context—that is, respectively, whether the thought occurs in spite of the presence of an external and often focal task or in the absence of such a task. The present study investigates the types of thoughts that arise in these contexts, what we call restricted and unrestricted mind wandering, using the presence or absence of a common thought-restricting task—a mindfulness task. Do the thoughts that emerge during restricted mind wandering differ from those that arise during unrestricted mind wandering?

### Restricted and unrestricted mind wandering

Mind wandering is often defined as arising in what we refer to as restricted contexts. That is, mind wandering is the unintentional shifting of attention away from a primary task to self-generated and task-unrelated thoughts (Smallwood & Schooler, 2006). Various terms such as unintentional thought, undirected thought, unguided attention, and perceptual decoupling have been used to emphasize mind wandering’s spontaneous and involuntary nature (see Barnett and Kaufman (2020); Blouin-Hudon and Zelenski (2016); Schooler et al. (2011); Irving 2016; Christoff 2012; Zedelius and Schooler (2016)). In contrast, others have emphasized the importance of thoughts arising in unrestricted contexts, such as in the case of Singer’s positive constructive daydreaming, which R. McMillan et al. (2013) described as “characterized by playful, wishful imagery, and planful, creative thought” (Dorsch, 2015; see also Klinger, 1978). Such thoughts are often considered deliberate and intentional cognitive activities, with individuals consciously selecting the focus and direction of their thoughts.

Restricted and unrestricted thoughts may diverge along various dimensions. For example, they may differ with respect to intentionality, deliberateness, and spontaneity (Christoff et al., 2016; Dorsch, 2015; Irving, 2016; Mills et al., 2018). However, to what extent restricted (e.g., mind wandering) or unrestricted (e.g., positive constructive daydreaming) thoughts fall into these categories is an area of active debate (Christoff et al., 2016; Dorsch, 2015; e.g., Irving, 2016; Murray & Krasich, 2020). Nonetheless, the objective distinction remains with respect to whether the thoughts arose in a restricted or unrestricted context. Our research therefore shifts the focus away from the contentious issues of intentionality and spontaneity, focuses instead on qualities of thoughts that arise in these various contexts. That is, we are concerned not with the origins of thoughts in restricted or unrestricted contexts, but with their content, aiming to understand the content and character of these thoughts in each context.

This distinction poses intriguing questions: What types of thoughts occur during episodes of restricted versus unrestricted contexts? For instance, using survey measures, some studies have suggested that engaging in unrestricted thought might lead to more positive emotions and enhanced life satisfaction (Brenner et al., 2022; Sugiura & Sugiura, 2020). In contrast, Killingsworth and Gilbert (2010) used experience sampling with an online app to probe people’s thoughts throughout the day. In this large-scale study involving several thousand participants, they found that ‘a wandering mind’ (i.e., restricted thoughts arising during another task) were a sign of an unhappy mind. Relevant to our current query, however, their approach—did not differentiate between restricted and unrestricted thought. In other words, it may be the case that both restricted and unrestricted thoughts are similarly negatively valenced. By explicitly examining thoughts that arise during restricted and unrestricted contexts, this present study aims to evaluate the qualities of these thoughts and elucidate their differences.

Specifically, the positivity of thought quality might vary depending on its temporal focus. Klinger (1978) observed that people’s minds wander through various topics, including worries and occurrences from the past, present, and future, as well as imaginary fantasies. Research has often found that restricted thoughts (so-called mind wandering) tends to be more future-oriented than the past-oriented (Smallwood & Schooler, 2015; Song & Wang, 2012; Stawarczyk et al., 2011). However, Kvavilashvili and Rummel (2020) makes a distinction between spontaneous future thinking—which may arise in restricted contexts—versus deliberate future thinking—which may arise during unrestricted contexts. Notably, future thinking is proposed to involve more cognitive effort and may therefore be more likely during unrestricted than restricted thought (Schacter et al., 2008; Smallwood et al., 2009). This has been further supported by evidence that individuals with higher working-memory capacity have more future-oriented task-unrelated thoughts (Baird et al., 2011). However, many of these studies rely on external judges to assess the future orientation of thoughts. In our study, we have participants self-assess their thoughts for future orientation.

Reality orientation is another aspect where restricted and unrestricted mind wandering might differ. Research suggests that off-task thoughts and imagination may activate similar brain regions (Villena-Gonzalez & Cosmelli, 2020). Moreover, the ability to guide imaginative thoughts has been linked with a greater likelihood of generating positive emotional outcomes (Holmes et al., 2016). This positive tendency is usually more evident in self-directed, unrestricted thought, where imagination is an actively engaged component. This distinction fits with the broader concept of voluntary thoughts, highlighting their capacity for meaningful insights and positive impacts, as discussed in the literature (McMillan et al., 2013).

Two common approaches to collecting thoughts in typical mind wandering research are self-caught and probe-caught (Schooler et al., 2011; Smallwood & Schooler, 2015): The self-caught method involves participants reporting their spontaneous thoughts, while the probe-caught method interrupts tasks to inquire if participants are currently engaged in spontaneous thought processes (e.g., Killingsworth & Gilbert, 2010). Because we are interested in capturing a rich representation of individuals’ thoughts over a short interval of time, we will focus on self-caught thoughts. This allows us to capture more fully formed and concrete thoughts, avoiding the potential issue of incomplete thoughts often associated with the probe-caught approach. It also allows us to comprehensively capture thoughts as they occur during a short time period. However, we note that probe-caught and self-caught methods differ in their findings (Schooler et al., 2004) and we do not argue that the self-caught approach can be generalized to probe-caught methods. Furthermore, while previous research has predominantly used the probe-caught method due to beliefs about individuals’ limited meta-awareness in reliably reporting spontaneous thoughts (Jackson & Balota, 2012; Schooler et al., 2004), our study focuses on the characteristic differences between thoughts in restricted and unrestricted contexts, allowing us to use the same method in both contexts. Recent studies by Varao-Sousa and Kingstone (2019) and Tortella-Feliu et al. (2020) suggest that individuals are quite capable of noticing and reporting their mind wandering experiences, indicating the viability of the self-caught method for our research objectives.

To compare restricted mind wandering with unrestricted mind wandering, we use tasks that either encourage participants to limit off-task thoughts or to think freely without constraints. For restricting thoughts, we implement a mindfulness task following previous research (Arch & Craske, 2006; Chu et al., 2023; Colzato et al., 2012; Vago & David, 2012). As described above, mind wandering typically occurs spontaneously, leading to a shift in attention. In contrast, mindfulness is about intentionally focusing attention away from spontaneous thoughts to the present moment (Girardeau et al., 2020). Belardi et al. (2022) has shown that mind wandering and mindfulness exist on opposite ends of a spectrum in terms of attention focus on the current moment (see also Mrazek et al., 2012). Therefore, contrary to the goals of previous researchers, we instruct participants to maintain a mindful state, which inherently involves suppressing any thoughts other than their focal point. Any deviation from this focus is classified as an unrestricted thought.

Additionally, this approach mitigates potential confounding factors related to working memory. Prior research has established that individual differences in working memory affect the frequency of task-unrelated thoughts (i.e., Kane & McVay, 2012; McVay & Kane, 2010; Mcvay & Kane, 2011; Robison & Unsworth, 2017). Levinson et al. (2012) found that using a breath-awareness task (a mindfulness task) did not result in differences in performance related to working memory capacity. Similarly, Meier (2019) reported no correlation between working memory and task-unrelated thoughts during a mindfulness task. These findings suggest that employing a mindfulness task helps control for the influence of individual differences in working memory on mind wandering. The current study will adopt this method and, in addition, we will ask participants to summarise their self-caught thoughts in a few words.

For the unrestricted mind wandering task, inspired by Dorsch (2015), participants will be encouraged to generate a series of self-directed thoughts, while withdrawing their attention from external stimuli. Instead of restricting their thoughts, participants will be instructed to freely explore their mental landscape, thinking anything that comes to mind, and then document each thought sequentially. This method enables the capture of a broad spectrum of naturally occurring thoughts.

The key distinction between restricted and unrestricted mind wandering in our study therefore lies in the nature of the tasks. Restricted mind wandering involves participants focusing on their breath, with the aim to directly inhibit thoughts and the linkage that may arise between one thought and the next. Conversely, in unrestricted mind wandering, there is an active directive to produce thoughts without inhibition. Participants are free to let their thoughts guide them, exploring their mental landscape without restrictions. Employing these distinct task contexts allows us to effectively study and compare the different types of thoughts that arise in restricted and unrestricted mind wandering.

Finally, to assess participants’ affects and understand how it influences different thought properties (i.e., Berntsen et al., 2015; Brenner et al., 2022; Marchetti et al., 2014; Poerio et al., 2013; Sugiura & Sugiura, 2020), we use the emotional recall task(ERT) and positive and negative affect scale (PANAS-SF). The ERT, designed by Li et al. (2020), requires participants to list recent emotions and rate them in terms of frequency and valence. The PANAS-SF, created by Watson et al. (1988), will be used to gain a more comprehensive picture of mood’s impact on the characteristics of thought.

In summary, to explore the distinctions between restricted and unrestricted mind wandering, we will evaluate thought characteristics in relation to valence, future orientation, and reality orientation. Further, we can compare thought qualities with participants’ affect measures.

## Methods

### Participants

A total of 96 (76 female, 20 male) first year psychology students participated in this study. Their mean age was 18.6 years (SD = 1.64) with a range of 17–33 years. Written consent was provided by the participant before testing in accordance with the University ethical consent policies. Each participant received course credit for participation. Data from 94 participants were analysed after excluding 2 participants who did not follow the instructions.

### Procedure

This study utilized a within-subjects design, where each participant engaged in both mindfulness and unrestricted mind wandering tasks, representing restricted and unrestricted mind wandering, respectively. The order of tasks was randomized, 47 beginning with the restricted mind wandering task and another 47 starting with the unrestricted mind wandering task. The experiment was conducted in-person using Qualtrics and took approximately 30–35 min.

Before the experiment started, participants were instructed to turn their phone to airplane mode and leave it outside the experiment room to prevent any distractions during the tasks.

For the restricted mind wandering (mindfulness) task, participants were asked to focus on their breath (instruction: As you inhale, think “inhaling” and as you exhale think “exhaling”). However, whenever they think of something other than their breath (mind wandering), they needed to use a short phrase to describe their intrusive thoughts and type it down, then press a blue arrow button to generate a new empty text box before refocusing back on their breath. They were informed the first task would last 10 min.

For the unrestricted mind wandering task, participants were told to clear their mind and then allow their mind to wander (instruction: you should allow your mind to wander freely, think whatever you like). Each time they think of something, they were instructed to use a short phrase to describe their intrusive thoughts and type it down, then press the blue arrow button to generate a new text box after they finish typing. Again, participants were told the task would last 10 min.

During both tasks, participants were told to fixate their eyes on the “+” at the centre of the screen to prevent distractions. After 10 min, the message “your time is up” appeared in red, letting them know they can press the forward button to begin the next task.

After each task, their typed thoughts were shown to the participants again, followed by these questions:

1. “Please rate how you feel about each of the thoughts you typed in. Was the thought unpleasant (1), pleasant (9), or somewhere in between.” (Likert scale: 1–9).
2. “Please rate how much the thought was related to the past, present or future? They were provided with a slider between 0 and 100 for each past, present, and future designation.” Thus, a thought could have 100 or 0 ratings for all three temporal designations, anything in between, or none at all (if all past, present and future were rated as “0”). For instance, thoughts without a temporal orientation might include pondering concepts like “do we have free will” or “pottery is made of clay”.
3. “Please rate to what extent your thought is related to something real vs something more imaginary. Real thoughts did happen or are likely to happen. Imaginary thoughts represent alternatives to real past events or future events that are more speculative. If something is neither imaginary or real, place the slider in the middle (50).” (Slider scale 0–100, 0 = imaginary 100 = real).

After completing the above tasks, participants completed the emotion recall task (Li et al., 2020): “Use 5 English words to describe feelings you have experienced during the past month.” Then participants were instructed to indicate how often they have experienced each of these feelings during the past month, with 0 indicating ‘not at all’ to 100 indicating ‘very often’. Finally, participants evaluated each of their feelings on the scale of unpleasant 1 to pleasant 9. If the feeling was completely neutral, neither unpleasant nor pleasant, participants were instructed to put 5. The ERT score was computed as $ERT=\frac{1}{500}\Sigma (rating - 5)*frequency$, producing a value between -4 and 4.

Participants then completed the PANAS questionnaire (Watson et al., 1988) (see appendix in https://osf.io/CT9Q7/) and provided their personal information: age and gender. The PANAS scores were computed by summing the 10 positive items(PA) and the 10 negative items(NA). A debriefing was presented after the experiment was finished, revealing the purpose of the current study.

### Dataset preparation

Given that the treatment order was randomized among participants, our initial analysis focused on whether the sequence of tasks (restricted versus unrestricted first) influenced total thought count. A variance test (F test) was conducted based on the number of thoughts generated in both conditions and the result (restricted mind wandering *F = 0.575*, unrestricted mind wandering *F = 0.704*) indicated that the variance of the two samples was similar. The order of the task did not significantly impact the number of thoughts produced.

Both ERT and PANAS results do not differ by treatment order. The Bayesian t-test results for ERT showed moderate evidence in favour of the null hypothesis $BF_{10} = 0.236\pm 0.02\%$. This indicated that task order did not influence the ERT results, with 4.24 times greater likelihood in support of the null. A Bayesian t-test found little support for differences based on treatment order in relation to PA from PANAS $BF_{10} = 0.226\pm 0.02\%$ or NA $BF_{10} = 0.357\pm 0.02\%$. Hence, both data sets were combined for subsequent analyses.

In order to compute time orientation (whether people were thinking about the past, present, or future), we use the following equation:

$$\begin{aligned} F=\frac{-1*past + 1*future}{past + present + future} \end{aligned}$$

where *past*, *present*, and *future* represent the slider amount assigned to each time orientation for each thought provided by the participant. The range is between –1 and 1, with values closer to 1 indicating future orientation, and values closer -1 indicating past thoughts. If the thought has a value near 0, it means that the thought is either present-oriented or past and future orientations were equally preferred. We also analysed each time orientation separately. There were 13 participant thoughts (across all participants) that had a 0 rating for each time orientation (past, present, future), and these were assigned a rating of 0. Therefore, a thought equal to 0 could also mean no time orientation.

### Statistical analysis

Our statistical analyses encompass both null hypothesis significance testing and Bayesian hypothesis testing. Where applicable, we employed linear mixed-effects models, treating participants’ IDs as random effects, using the lme4 package (Bates et al., 2015). In parallel, we also present the Bayesian multi-level equivalents and provide the 95% credible intervals, utilizing the brms package (Bürkner, 2017, 2021). All of our code and data are freely available on Open Science Framework (https://osf.io/CT9Q7/).

## Results

### People generate more thoughts in unrestricted than restricted condition

**Fig. 1:**
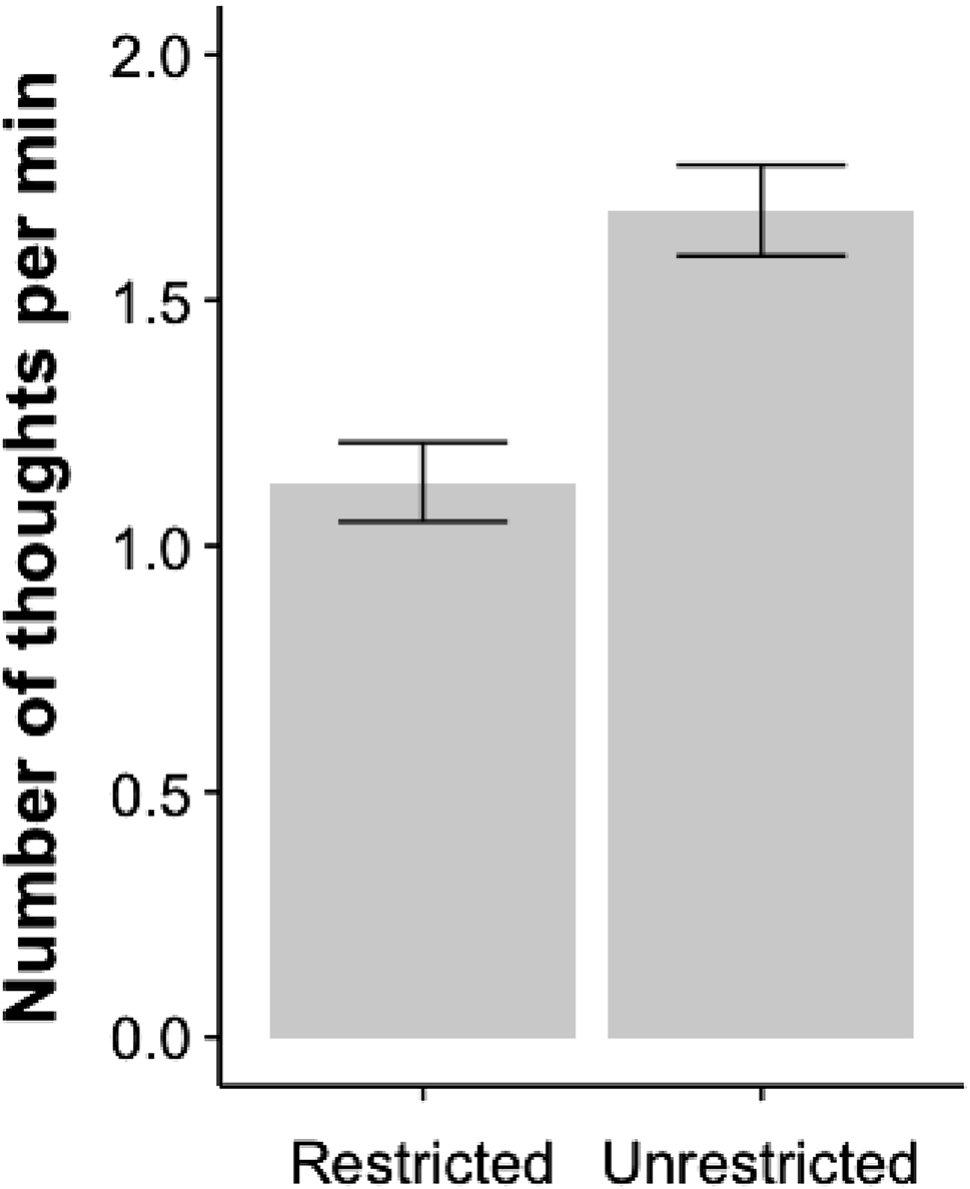
Thoughts per minute during restricted (mindfulness task) and unrestricted(free thought task) mind wandering. *Error bars equal standard error of mean*

More thoughts were produced during the unrestricted mind wandering task compared to the restricted mind wandering task. Within a 10-minute period, 1582 thoughts were recorded during unrestricted mind wandering, while 1062 thoughts emerged during restricted mind wandering. On average, 1.13 thoughts per minute were generated during restricted mind wandering task ($M=11.30$, $SD=7.83$), while 1.68 thoughts per minute were generated during unrestricted mind wandering task ($M=16.83$, $SD=8.98$) (see Fig. 1), $t(93)=-7.204, p<0.000$. These results not only served as a manipulation check but also indicate that spontaneous thoughts still occur surprisingly often even during a mindfulness task aimed at deliberately inhibiting distracting thoughts.

### The Restricted mind is less happy than the unrestricted mind

**Fig. 2:**
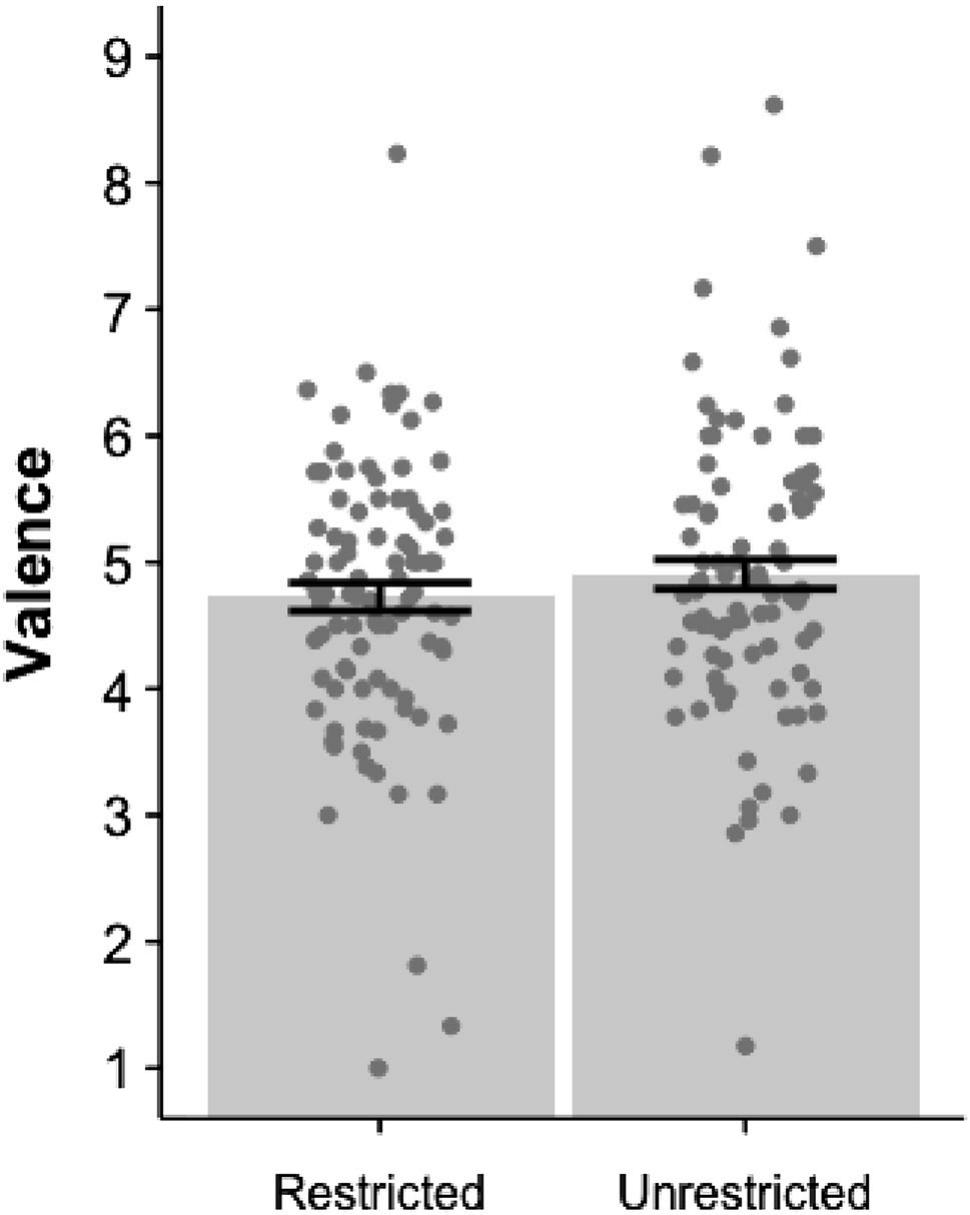
Mean valence for thoughts produced during restricted (mindfulness task) and unrestricted(free thought task) mind wandering. *Error bars equal standard error of mean*

The majority of thoughts were unpleasant. Our data showed that during restricted mind wandering, on average, 45.50% of thoughts were unpleasant (rated < 5), while only 28.18% were pleasant (rated > 5). Unrestricted mind wandering showed a similar pattern, with a tendency to think more unpleasant thoughts (42.97%) than pleasant thoughts (36.08%) (see Fig. 2).

Comparing the two treatments, thoughts occurring during the unrestricted condition had a higher valence ($M=4.91$, $SD=1.88$) than those occurring in the restricted condition ($M=4.73$, $SD=1.71$). A linear mixed effects analysis, with participants’ ID entered as a random effect, indicated higher valence thoughts for the unrestricted condition than restricted condition, $\beta {_{unrestricted(1)}=0.2003},SE = 0.08,X^2(1) = 6.25, p=0.01, Cohen's d{_{unrestricted(1)}=0.1}$. The Bayesian multilevel model further supported these results, $\beta {_{unrestricted(1)}=0.20},95\%CrI = [0.05, 0.36]$ (Note: CrI indicates the 95% credible interval; a non-overlapping 0 indicates Bayesian support for the alternative hypothesis). We note the effect sizes are fairly small here, suggesting that the differences, though statistically supported, are less notable than the similarities. In addition, the valence of thoughts produced during restricted and unrestricted mind wandering were positively correlated ($r(92)=0.505$, $p<0.001$, $BF_{10} = 81056.54\pm 0\%$).

### Unrestricted thoughts are more future oriented and more positive than restricted thoughts

**Fig. 3:**
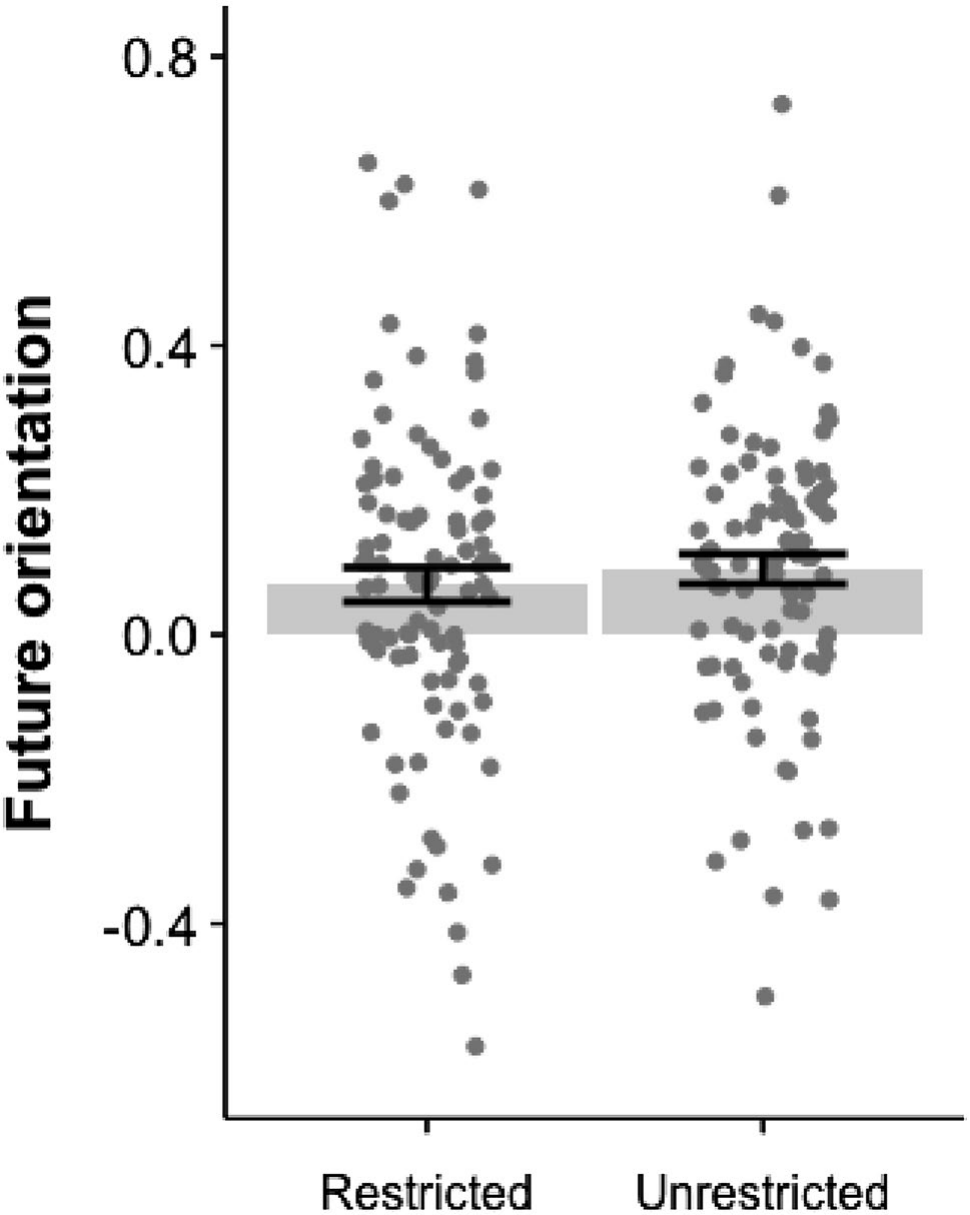
Future orientation of thoughts produced during Restricted (mindfulness task) and Unrestricted(free thought task) mind wandering. *Error bars equal standard error of mean*

Both unrestricted and restricted thoughts show a tendency towards future orientation, but unrestricted mind wandering is more future oriented ($M=0.091$, $SD=0.201$) than restricted mind wandering ($M=0.079$, $SD=0.229$). Moreover, unrestricted mind wandering produced a greater proportion of future-oriented thoughts (45.4%) compared to restricted mind wandering (43.1%), considering thoughts with a future orientation score above 0, $t(93)=-5.37, p<0.000$. This is consistent with past work on restricted mind wandering (Smallwood & Schooler, 2015; Song & Wang, 2012; Stawarczyk et al., 2011), but further suggests that unrestricted thoughts may be still more inclined towards future thinking (see Fig. 3). The linear mixed effects analysis, with task as the fixed effect and participants’ IDs as the random effect, revealed that during unrestricted mind wandering, people’s thoughts were more future-oriented compared to restricted mind wandering ($\beta {_{unrestricted(1)}=0.036},SE = 0.017,X^2(1) = 4.49, p=0.03, Cohen's d{_{unrestricted(1)}=0.08}$). The Bayesian multilevel model supports this finding ($\beta {_{unrestricted(1)}=0.04},95\%CrI = [0.002, 0.068]$). Again, though the effect sizes are small and emphasize overall similarity, the result suggests that unrestricted mind wandering is associated with a higher degree of future orientation.

Similar results are found by assessing each temporal orientation measure independently. A linear mixed-effects analysis—with task as the fixed effect and participants’ IDs as the random effect—finds the following: Compared with restricted mind wandering, unrestricted mind wandering is

- Slightly past oriented($\beta {_{unrestricted(1)}=3.726},SE = 1.431,X^2(1) = 6.78, p=0.009, Cohen's d{_{unrestricted(1)}=0.1}$. The Bayesian multilevel model provides evidence for such an effect $\beta {_{unrestricted(1)}=3.74},95\%CrI = [0.86, 6.61]$);
- Less present oriented ($\beta {_{unrestricted(1)}=-2.835},SE = 1.329,X^2(1) = 4.55, p=0.03, Cohen's d{_{unrestricted(1)}=-0.08}$. The Bayesian multilevel model provides evidence for such an effect $\beta {_{unrestricted(1)}=-2.82},95\%CrI = [-5.42, -0.23]$);
- More future oriented ($\beta {_{unrestricted(1)}=7.877},SE = 1.520,X^2(1) = 4.55, p<0.001, Cohen's d{_{unrestricted(1)}=0.2}$. The Bayesian multilevel model provides evidence for such an effect $\beta {_{unrestricted(1)}=7.90},95\%CrI = [4.97, 10.92]$).

### Future orientation is the happiest orientation

Future thoughts tended to be more positively valenced than present or past thoughts. The results of a linear mixed effects analysis, with time orientation score as the fixed effect and participants’ IDs as the random effect, found that an increase in future thinking is associated with more pleasant thoughts ($\beta {_{future orientation}=0.198},SE = 0.091,X^2(1) = 4.70, p=0.03, Cohen's d{_{future orientation}=0.08}$). The Bayesian multilevel model supported these findings, providing evidence for such an effect, $\beta {_{future oreientation}=0.20},95\%CrI = [0.02, 0.37]$.

Further analysis of each temporal orientation measure individually, with the score as the fixed effect and participants’ IDs as the random effect, revealed the following:

- Past thoughts did not influence valence ($p>0.05$).
- Present thought were negatively associated with valence ($\beta {_{present}=-0.01},SE = 0.001,X^2 = 65.47, p<0.0001, Cohen's d{_{present}=-0.31}$. The Bayesian multilevel model provides evidence for such an effect $\beta {_{present}=-0.009},95\%CrI = [-0.01, -0.007]$);
- Future thought were positively associated with valence ($\beta {_{future}=0.003},SE = 0.001,X^2 = 9.82, p=0.002, Cohen's d{_{future}=0.12}$. The Bayesian multilevel model corroborates this finding $\beta {_{future}=0.003},95\%CrI = [0.001, 0.005]$).

### Reality orientation influences future orientation but not valence

Reality orientation was similar for restricted and unrestricted mind wandering, with both showing approximately a quarter of all thoughts being dominantly imaginary: imaginary thoughts (restricted: 22.32%; unrestricted: 24.21%) and real (restricted 72.72%; unrestricted 69.61%), when considering thoughts rated above and below 50 on the reality orientation slider. Results of a linear mixed effects analysis with task as the fixed effect and participants’ IDs as the random effect were insignificant ($p>0.05$). The Bayesian hypothesis test further supports this finding with evidence in favour of the null hypothesis $BF_{10} = 0.221\pm 0.08\%$.

**Fig. 4:**
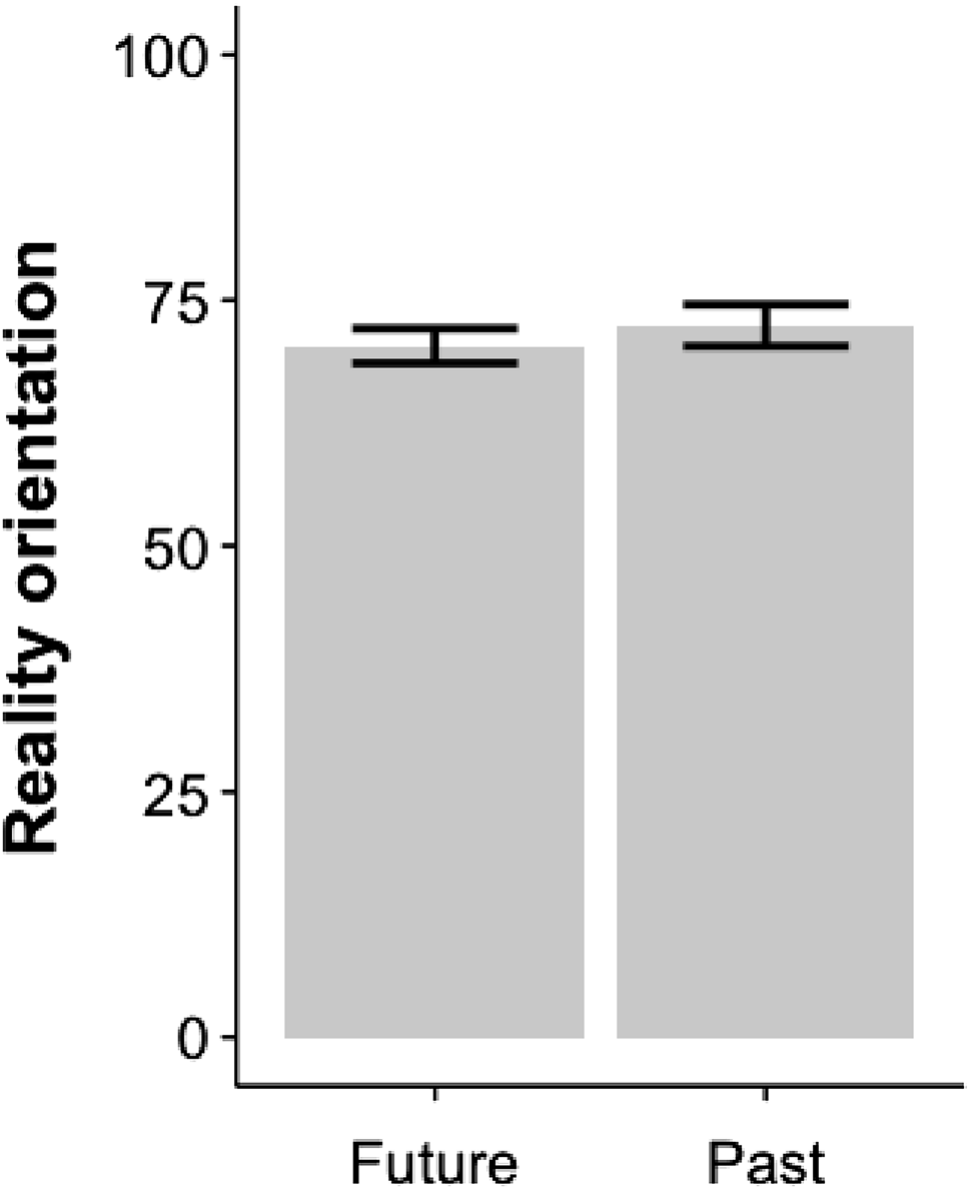
Reality orientation of thoughts produced during Restricted (mindfulness task) and Unrestricted(free thought task) mind wandering. *Error bars equal standard error of mean*

Our linear mixed effects analysis with future orientation score as the fixed effect and participants’ IDs as the random effect, we found that future orientation is negatively correlated with reality orientation ($\beta {_{futureorientation}=-3.770},SE = 1.386,X^2(1) = 7.3889, p=0.007, Cohen's d{_{futureorientation}=-0.11}$). The Bayesian multilevel model also supported these findings, $\beta {_{future oreientation}=-3.75},95\%CrI = [-6.50, -1.05]$. This suggests that an increase in future thinking is associated with more imaginary thoughts. This is consistent with the results shown in Fig. 4, where the mean reality orientation score for future thoughts ($M = 70.35$, $SD = 12.77$) is lower than that for past thoughts ($M= 72.44$, $SD = 12.53$).

However, when analysing each temporal orientation score individually, with the score as the fixed effect and participants’ IDs as the random effect, we further found that:

- Past thoughts did not significantly influence the reality orientation ($p>0.05$).
- Present thoughts showed a positive association with reality orientation ($\beta {_{present}=0.042},SE = 0.018,X^2 = 5.41, p=0.02, Cohen's d{_{present}=0.09}$. This is supported by the Bayesian multilevel model $\beta {_{present}=0.041},95\%CrI = [0.005, 0.076]$);
- Future thought were negatively associated with reality orientation ($\beta {_{future}=-0.038},SE = 0.016,X^2 = 6.11, p=0.01, Cohen's d{_{future}= -0.09}$, as evidenced by the Bayesian multilevel model $\beta {_{future}=-0.038},95\%CrI = [-0.069, -0.007]$).

Overall, this reveals that a higher future orientation score generally corresponds to less realism. Specifically, future thoughts tend to be construed as more imaginary, and present thoughts tend to be construed as more real. However, the past alone does not show a similar effect and has no predictive relationship with realism. This implies that the relationship between future orientation and realism is not influenced by past thoughts (past thoughts can be both real or imaginary).

A linear mixed effects analyses found that valence did not significantly associate with reality orientation ($p>0.05$). This indicates that imaginary thoughts are not inherently more or less positive/negative than real thoughts. Interestingly, when focusing on instances of real thoughts (rating = 100), we observed a more positive valence for unrestricted thought compared to mind wandering ($\beta {_{unrestricted(1)}=0.601},SE = 0.149,X^2(1) = 16.26, p<0.000, Cohen's d{_{unrestricted(1)}=0.27}$). The Bayesian multilevel model also supported these findings $\beta {_{unrestricted(1)}=0.60},95\%CrI = [0.31, 0.88]$. These results suggest that when experiencing real thoughts, individuals engaged in unrestricted mind wandering tend to have more positive thoughts than those engaged in restricted mind wandering.

### Affect influences thought valence and reality orientation

Table 1 summarizes the results regarding the influence of the affect measures on thought quality for restricted and unrestricted mind wandering. The results include outcomes from a linear regression analysis as well as a corresponding Bayesian regression.

Firstly, affect does not predict participants’ future orientation scores. Secondly, both the ERT and PANAS are predictors of participants’ valence scores. Specifically, the ERT and PA are positively correlated with valence, while NA is negatively correlated with valence in both restricted and unrestricted mind-wandering. As for reality orientation, only NA predicts the score in both types of thought, showing a negative correlation. Finally, in the case of unrestricted mind wandering, only the ERT exhibits a positive correlation with reality orientation.

**Table 1:** Affect influences thought valence and reality orientation, but not time orientation

Thought Type	beta	SE	F	R$^2$	p	BF10
Restricted Mind wandering mean valence						
ERT	0.429	0.108	15.76	0.15	0.000*	162.04
PA	0.045	0.015	8.73	0.09	0.004*	9.27
NA	$-$0.047	0.015	9.79	0.1	0.002*	14.37
Unrestricted Mind wandering mean valence						
ERT	0.518	0.11	22.4	0.2	0.000*	2114.12
PA	0.047	0.016	8.62	0.09	0.004*	8.83
NA	$-$0.049	0.016	9.76	0.1	0.002*	14.20
Restricted Mind wandering reality orientation						
ERT	0.501	1.857	0.07	0.00	0.788	0.224
PA	0.08	0.254	0.1	0.00	0.754	0.226
NA	$-$0.798	0.239	11.14	0.11	0.001*	25.14
Unrestricted Mind wandering reality orientation						
ERT	3.486	1.717	4.12	0.04	0.045*	1.311
PA	0.325	0.238	1.86	0.02	0.176	0.491
NA	$-$0.961	0.217	19.53	0.18	0.000*	708.07
Restricted Mind wandering time orientation						
ERT	$-$0.007	0.025	0.09	0.00	0.764	0.225
PA	0.002	0.003	0.21	0.00	0.651	0.237
NA	0.002	0.003	0.29	0.00	0.592	0.246
Unrestricted Mind wandering time orientation						
ERT	$-$0.024	0.023	1.22	0.01	0.272	0.371
PA	0.001	0.003	0.04	0.00	0.845	0.220
NA	0.002	0.003	0.40	0.00	0.527	0.259

## Discussion

Life is a sea of thought, in both restricted and unrestricted contexts. Do these contexts generate different kinds of thought? The present research makes several novel contributions to answering this question. First, people generate more thoughts—by a factor of approximately 2—during unrestricted than restricted mind wandering. While thoughts in restricted mind wandering were still frequent (occurring more than once per minute), they were less abundant than in unrestricted mind wandering. Second, thoughts were generally more negative than positive in both restricted and unrestricted contexts, in line with previous studies (e.g., Killingsworth & Gilbert, 2010), thoughts during unrestricted mind wandering were more positively valenced than those during restricted mind wandering. This supports Klesel et al. (2021) finding that deliberate mind wandering, similar to unrestricted thought, is more often associated with enjoyment—even if only relatively so. Third, thoughts produced during unrestricted mind wandering were more future oriented, and future oriented thoughts were more positively valenced. This supports Singer (1975)‘s control-based theory, proposing that self-directed thoughts can be positively guided. Collectively, we conclude that unrestricted mind wandering is more positively valenced than mind wandering, potentially due to its inclination to produce more future-oriented thoughts. Fourth, despite the many nuanced differences described above, effect sizes were dominantly small across the board, suggesting that thoughts that arise during restricted and unrestricted mind wandering are more similar than different. Thus, even if the many difficult to operationalize concepts—such as ’deliberate’, ‘intentional’, and ‘spontaneous’—differ in the two contexts, the kinds of thoughts they tend to generate are highly similar.

Our results also contribute to better understanding the proportion and contribution of imaginary thought in restricted and unrestricted contexts. Imaginary thoughts constitute approximately one-quarter of all thoughts, with no notable difference between restricted and unrestricted mind wandering. That is, on average, about every fourth thought is speculative, meaning that it is not drawn from memory of a specific experience, but composed of more generative constructions of a counter-factual or exploratory nature. This is exciting, as such thoughts are known to be instrumental to human cognitive exploration (Hills, 2019; Suddendorf, Thomas et al., 2022). Our research suggests that they occur approximately once every two to four minutes.

Contrary to the findings of Stawarczyk et al. (2011), which suggested that future-oriented mind wandering was perceived as more realistic and concrete, our results indicate that an increase in future thinking is associated with a greater number of imaginary thoughts. Additionally, we found that the reality orientation of thoughts did not appear to influence their valence. Interestingly, when focusing solely on 100% reality-oriented thoughts, those engaged in unrestricted mind wandering tended to have happier thoughts compared to those in restricted mind-wandering. It is important to note that the effect sizes for all these observations were small as well.

Echoing the findings of Andrews-Hanna et al. (2013), thought valence is predictably correlated with the ERT and PANAS. More positive state-level affect was associated with more positively valenced thoughts. Surprisingly, we found no evidence supporting a connection between future orientation and ERT or PANAS. This may indicate that future oriented thoughts are not directly related to state-level affect. On the other hand, NA was negatively correlated with reality orientation in both restricted and unrestricted mind wandering. This implies that in both forms of mind wandering, a more negative mood associated with increased imaginative thinking. Given that future oriented thoughts were more positively valenced, engaging in more future orientated real thought may therefore help reduce negative affect (as suggested by Blouin-Hudon & Zelenski, 2016).

To summarize, our study distinguishes the cognitive and emotional dynamics between restricted and unrestricted mind wandering. We discovered that unrestricted mind wandering is generally more positively valenced and future-oriented compared to restricted mind wandering. Although more thoughts are generated when individuals control their thought processes, the ratio of imaginative to real thoughts does not significantly differ between the two types of thinking. However, the small effect sizes observed in our results suggest an essential similarity between restricted and unrestricted mind wandering.

### Limitation and clarification

While our research significantly advances the understanding of the similarities and differences between restricted and unrestricted mind wandering, it is important to recognize certain limitations for future research considerations. One major concern involves the retrospective rating of thoughts by participants, which we introduced to allow participants to produce thoughts with limited interruption. However, as Kahneman (2011) has suggested, this process might be more influenced by participants’ experiences during the rating rather than their experiences at the time the thought occurred. Additionally, Gonzalez-Castillo et al. (2021)’s research indicates that retrospection is subject to inaccuracies in episodic memory. Critically, our study employed a within-subject design, ensuring that the retrospective nature of the rating process was consistent across both tasks.

Another limitation of our research and mind wandering research more broadly is the challenge of determining whether thoughts arising in restricted or unrestricted contexts are “self-guided”, “intentional”, “deliberate”, or even “off-task” (e.g., if the participant decides the primary task is less important than some other obligation they must plan for outside the laboratory). This is a controversial area with many notable researchers weighing in on either side (Irving, 2016; Murray & Krasich, 2020; Seli & Risko2016a; e.g., Seli & Schacter, 2016b; Seli et al., 2018). Our research is not designed to address this question, but it does nonetheless speak to the apparent division. If we take the mindfulness paradigm as indicative of producing thoughts commonly considered to be off-task mind wandering, and our unrestricted paradigm as indicative of more consciously self-guided and deliberate thought, then our results suggest that despite the potential differences in origins among these thoughts, they are nonetheless highly similar.

Finally, our participants may have perceived the recording of non-task-related thoughts as a ‘penalty’ in the mindfulness task, leading them to disregard some thoughts. This would place our estimate of thought count as a lower bound, as they might have produced more thoughts. Unless the thoughts are otherwise differentiated by this ‘penalty’, our remaining findings stand.

## Data Availability

The datasets used and the R code for analysis are available in the OSF (https://osf.io/CT9Q7/).
